# Protein Identification of Venoms of the African Spitting Cobras, *Naja mossambica* and *Naja nigricincta nigricincta*

**DOI:** 10.3390/toxins12080520

**Published:** 2020-08-14

**Authors:** Ottilie Katali, Loide Shipingana, Peter Nyarangó, Mirva Pääkkönen, Erastus Haindongo, Timothy Rennie, Peter James, John Eriksson, Christian John Hunter

**Affiliations:** 1 Faculty of Health Sciences, University of Namibia, Windhoek 9000, Namibia; ottiliekatali@gmail.com (O.K.); loideshipingana@gmail.com (L.S.); pnyarango@gmail.com (P.N.); ehaindongo@unam.na (E.H.); trennie@unam.na (T.R.); 2 Turku Bioscience Centre, University of Turku and Åbo Akademi University, FI-20520 Turku, Finland; mirva.paakkonen@bioscience.fi (M.P.); peter.james@immun.lth.se (P.J.); John.Eriksson@abo.fi (J.E.); 3 Cell Biology, Faculty of Science and Technology, Åbo Akademi University, FI-20520 Turku, Finland

**Keywords:** African spitting Cobras, *Naja mossambica*, *Naja nigricincta nigricincta*, snake venom, snakebites, antivenin

## Abstract

Cobra snakes, including *Naja mossambica* and *Naja nigricincta nigricincta,* are one of the major groups of snakes responsible for snakebites in southern Africa, producing significant cytotoxicity and tissue damage. The venom of *N. mossambica* has been briefly characterised, but that of *N. n. nigricincta* is not reported. The current study identifies the venom proteins of *N. mossambica* and *N. n. nigricincta*. This is achieved using sodium dodecyl sulphate (SDS)-polyacrylamide gel eletrophroresis (PAGE), followed by high-performance liquid chromatography-tandem mass spectrometry (HPLC-MS/MS). Most of the proteins were less than 17 kDa in both snakes. *N. mossambica* was found to have 75 proteins in total (from 16 protein families), whereas *N.n. nigricincta* had 73 (from 16 protein families). Of these identified proteins, 57 were common in both snakes. The proteins identified belonged to various families, including the three-finger toxins (3FTx), Cysteine-rich secretory proteins (CRiSP), Phospholipase A2 (PLA_2_) and Venom metalloproteinase M12B (SVMP). The current study contributes to the profile knowledge of snake venom compositions, which is of fundamental value in understanding the proteins that play a major role in envenomation.

## 1. Introduction

**Figure 1:**
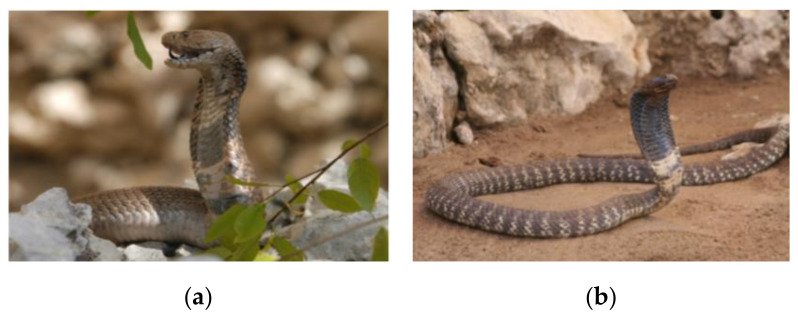
The figure shows two common spitting cobras in Namibia under study: (**a**) *Naja mossambica* (Mozambique spitting cobra); (**b**) *Naja nigricincta nigricincta* (western barred spitting cobra or zebra snake). Photos courtesy of the Living Desert Snake Park (Angela Curtis and Stretch Cowbrink).

Snakebites are a major cause of morbidity and mortality in Africa, Asia and Latin America, [1,2], yet, a neglected tropical disease—mostly affecting remote and rural tropical regions [3,4,5]. The burden of snakebites has been estimated at 1.2 to 5.5 million per year worldwide [6], with about 400,000 victims ending up with permanent disabilities [2,7]. In Namibia, Southern Africa, 721 snakebites were recorded over the course of 12 months (August 2015 to July 2016) in the main referral hospital in the capital city, Windhoek [8]. These snakebites were caused by different snakes, including spitting cobra snakes, and a large proportion of the people bitten were children under the age of six-years-old—and many of these children suffered long-term disability [8]. Medically important Namibian spitting cobras include the *Naja mossambica* (Mozambique spitting cobra,) *N. nigricollis* (black-necked spitting cobra) and the *N. nigricincta nigricincta* (western barred spitting cobra or zebra snake), and its subspecies of *N. nigricincta woodi* (black spitting cobra) [9,10,11,12] (Figure 1). *N. n. nigricincta* is the most problematic.

The limitation of medical facilities in rural areas makes it a challenge for snakebite victims who are often several hours away from the nearest health centre, and may not be equipped to effectively manage these medical emergencies [13,14]. Snakebite management is challenging as the identities of the snakes is not always known [15], and there is incomplete knowledge about best treatment practices. The choice of treatment in Namibia is usually limited to (1) supportive care, (2) a monovalent antivenin for confirmed *Boomslang* bites and (3) a polyvalent antivenin from the South African Institute for Medical Research (SAIMR) for any other snakebites [16].

The efficacy of the antivenins is highly dependent on the snake venom from which it is developed [17]. The SAIMR polyvalent antivenin is based on the venoms from ten snakes of viperine and elapids families (South African Vaccine Producers (PTY) Ltd., Gauteng, South Africa), and therefore, thought to be effective against those respective snakes [18,19,20,21]. However, the *N. n. nigricincta* venom is not included the production of this polyvalent. Locally, it has been observed that the polyvalent seem ineffective against the *N. n. nigricincta,* of which some local clinicians indicated that aggressive debridement is the cornerstone of snakebite management.

In general, envenomations from these snakes cause severe cytotoxicity, necrosis, haemorrhage, anticoagulant and thrombolytic injuries, which may result in death [19]. These potent characteristics of envenomations are due to the venom compositions of the snakes. In general, the lyophilised venom compositions of numerous snakes are made up of 80% to 100% proteins and other non-protein components, including carbohydrates, lipids, amines, and inorganic salts [22]. Table 1 summarises the common protein families in various snake venoms and their functions. In elapids, the venom compositions mainly consist of two protein families: (1) Three-finger toxins (3TFxs), which are in highest abundance in most species and (2) the phospholipase A2s (PLA2s), which are the second most abundant [22]. The lyophilised venom composition of the *N. mossambica* from Tanzania has been characterised and is made up of 99% proteins, where ~69% are 3TFxs, ~27% are PLA2s, and ~3% are the snake venom serine proteases (SVSPs) [23]. The other ~1% of the venom is made up of endonuclease and unknown substances [23]. To our knowledge, the venom composition *N. n. nigricincta* has not been characterised. However, in animals, the *N. n. nigricincta* venom has been reported to possess haemorrhagic, necrotic, procoagulant and thrombolytic activities [13]. The present study aimed to profile the venom composition of *N. mossambica* and *N. n. nigricincta* from Namibia.

## 2. Results

**Figure 2:**
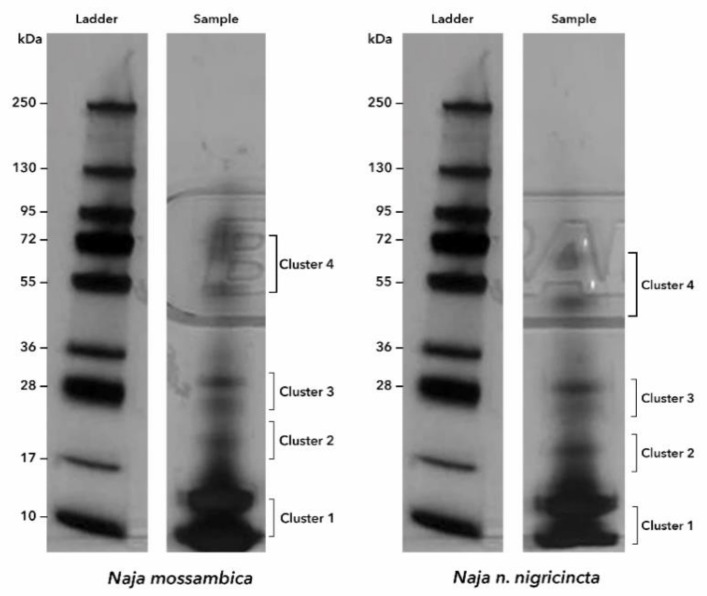
This figure shows the SDS-PAGE of protein separation of the venoms of *N. mossambica* and *N. n. nigricincta.* The molecular weight marker (in kDa) is on the left of each venom sample for *N. mossambica* and *N. n. nigricincta.* The proteins were run in duplicates at 200 V for 40 min until the dye front reached the end of the six to 16% precast gel. The gels were silver-stained and digested with trypsin and analysed with HPLC-MS/MS.

The sodium dodecyl sulphate (SDS)-polyacrylamide gel eletrophroresis (PAGE) results indicated that most of the proteins were less than 17 kDa in both *N. mossambica* and *N.n. nigricincta*, as shown in cluster one (Figure 2).

**Figure 3:**
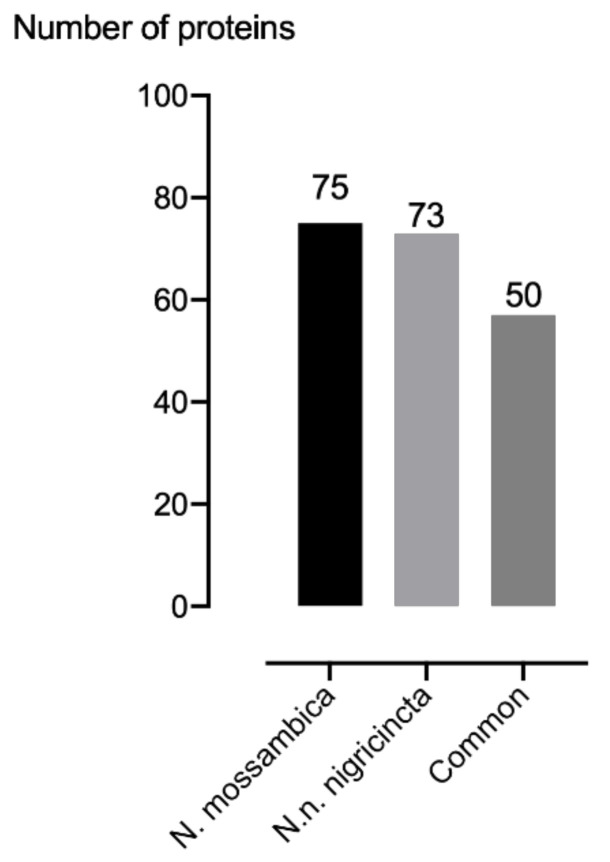
This figure shows the protein counts for *N. mossambica* and *N.n. ningricincta* venom and those common to both snakes.

The high-performance liquid chromatography-tandem mass spectrometry (HPLC-MS/MS) raw data files were searched with Proteome Discoverer 2.2 software (Thermo Fisher Scientific, Waltham, MA, USA). Proteins which were identified with only one peptide were filtered out from the results. Only four and three proteins were identified when the *N. mossambica* and *N. n. nigricincta* venom samples were analysed without digestion, respectively. A total of 32 and 22 proteins, from *N. mossambica* and *N. n. nigricincta* respectively, were identified with in-solution digestion. However, the vast majority of the proteins were identified from in-gel digested samples (see Table 2). When all approaches were merged, a total of 75 and 73 proteins were identified for *N. mossambica* and *N. n. nigricincta* which belong to 16 families per snake. Of these, 57 were common to both snakes (see Figure 3 and Table 3). The mass of the proteins identified ranged from Individual 2.7 to 184.6 and 3.4 to 184.6 kDa for *N. mossambica* and *N.n. nigricincta* respectively (See Tables S1–S3). The quantity of the identified proteins was not determined.

Three-finger toxins (3FTx) seemed to be the most abundant protein family, with 30 and 25 identified in *N. mossambica* and *N. n. nigricincta,* respectively. Various proteins were identified from the following families: Cysteine-rich secretory proteins (CRiSP), Phospholipase A2 (PLA_2_) and Venom metalloproteinase M12B (SVMP). Unique protein families identified in *N. mossambica* included cathelicidin and venom kunitz-type. On the other hand, glycosyl hydrolase 56 and peroxiredoxin families were only found in *N.n. nigricincta* (See Table 3 and Table S1).

Prior to HPLC-MS/MS analysis, proteins were either subjected to silver stain in-gel digestion, (ii) no stain in-gel digestion, (iii) in-solution digestion, and (iv) no digestion. The percentage is relative to the total number of proteins from all approaches merged for *N. mossambica* (75 proteins) and *N. n. nigricincta* (73 proteins).

## 3. Discussion

The venoms of the cobra snakes *N. mossambica* and *N.n. nigricincta* are known to possess cytotoxic activity to their victims [13,19]—*N. nigricincta* is also reported to possess haemorrhagic, anticoagulant, and thrombolyt activity in animals [13]. The present study aimed to characterise the venoms of these cobra snakes in order to identify the specific proteins present. We carried out a bottom-up approach, however, acetonitrile precipitated proteins were analysed without digestion, in order to identify the protein complexity of the venoms of *N. mossambica* and *N. n. Nigricincta.* The protein complexity of the venoms of *N. mossambica* from Tanzania was previously characterised [23], while that of *N. n. nigricincta,* to our knowledge, is reported for the first time in the present study.

In the current study, it is demonstrated that various proteins known to be responsible for pathological effects reported in various snake envenomation are also present in the two snakes, *N. mossambica* and *N. n. nigricincta* [22]. Moreover, the venom of the two snakes share more than 50% of the identified proteins. The majority of these proteins had a molecular mass of about 17 kDa and less (see Figure 2). Most proteins seemed to belong to the 3FTx family followed by CRiSP, SVMP and PLA_2_ and a couple from other families (see Table 3 and Table S1). However, this is the first study on the venom profile of *N. n. nigricincta*, similar protein families were reported by Petras et al. [23] for the venom of *N. mossambica* with the exemption of endonuclease family, of which our current study did not record. In addition, 12 more other protein families were identified for *N. mossambica* in the present study (Table 3). The variation among studies in protein identification may be due to the difference in the geographical location of the snakes. Moreover, Petras et al. [23] identified proteins from the Nawaprin family in the venom of *N. nigricollis* from Nigeria, Togo, and Cameroon, but not in that of the Tanzanian, which again may be due to different geographical locations.

Due to the complexity of snake venom proteins, only a fraction of proteins and peptides can be identified in a single run of LC-MS/MS [40,41]. Hence, the experimentation of such complex samples is very critical, and the differences in techniques may result in variations among the analysis. In order to produce a high-throughput protein identification. The current study used four sample preparation methods incorporated with HPLC-MS/MS analysis (see Table 2), of which each method gave only a fraction of the total proteins identified. For instance, in *N. n. nigricincta,* the fragment cysteine-rich venom protein mossambin (accession, P0DL16) and cytotoxin Vc-5 (accession, Q9PS34) were only identified with the in-solution digestion approach. Whereas, basic phospholipase A2 nigexine (accession, P14556) was only identified with the silver-stained in-gel digestion method (See Table S4).

Furthermore, the precursor ion exclusion list consisting of all identified proteins in preceding runs was used to analyse HPLC-MS/MS analysis output for the no stain in-gel digestion method. This method eliminated redundant proteins of high abundance, allowing identification of new and unique proteins that were missed in the first run. For example, c-type lectin galactose-binding isoform (accession, D2YVI2) and cytotoxin SP13b (accession, P60306) were identified only after the subjection of the exclusion list. Thus, the use of different sample preparation methods and replicated runs, coupled with the exclusion list, improved the comprehensiveness of protein analysis (See Table S3).

There have been several monovalent and polyvalent antivenins used for cross neutralisation of snake envenomations [18,42]. The efficiency of the antivenin is highly dependent on the snake venom, on which it is based [17]. The South African Institute for Medical Research (SAIMR) polyvalent antivenin is the most commonly used in Africa [16,18], which has been used for cross neutralisation of the venom for more than 35 years [19]. In Namibia, most *N.n. nigricincta* envenomation incidences treated with this antivenin have been recorded to undergo radical excision around the site of the snakebite [43]. The treatment of these envenomations is done using the SAIMR polyvalent [16], of which *N.n. nigricincta* is not considered in its preparation [18,43]. Another example of ineffective antivenin has been reported by Sintiprungrat et al. [13], where the antivenin produced against the Indian *N. naja* was less effective against the Sri Lankan *N. naja.* The composition of the venom from these species has geographical intra-specific variations [17].

## 4. Conclusions

In conclusion, even though the composition of the proteins was not quantified, the venoms of the two cobra snakes *N. mossambica* and *N.n. nigricincta* have been successfully profiled, revealing a vast spectrum of proteins. The current study proposes further characterisations of these venoms for quantification to better understand these proteins, their toxicity and even therapeutic candidacy.

## 5. Materials and Methods

### 5.1. Snake Venom Samples

The venoms were milked from each adult *N. mossambica and N.n. nigricincta* and were lyophilised, yielding 95.3 and 129.2 mg, respectively [13]. These crude venom samples were stored at −20 °C until experimentation. The study was approved by National Commission on Research Science and Technology of Namibia through the University of Namibia (Certificate: RCIV00022018) and the exportation of samples to the Turku Bioscience Centre, Finland was permitted by the Namibian Ministry of Environment and Tourism (Permit: 118430).

### 5.2. Venom Protein Separation by SDS-PAGE and In-Gel Digestion

Crude venoms were dissolved in Laemmli buffer (one mg in 200 µL). From these samples, 50 µL were then separated by SDS-PAGE in six to 16% precast gel (Bio-Rad, Hercules, CA, USA). This was run at 200 V for 40 min until the dye front reached the end of the gel, was silver-stained, and cut into 16 pieces for a lane. Another gel was run under similar conditions except it was only allowed to run for 5 cm into the resolving gel and was not stained. These gels were digested using trypsin enzyme as described previously [44,45,46].

### 5.3. Acetonitrile Precipitation and In-Solution Digestion

Proteins (>10 kDa) were removed from the venom samples by acetonitrile precipitation. One mg of a dry venom sample was dissolved in 200 µl 80% acetonitrile, and then centrifuged for six minutes at 5000 relative centrifugal force (rcf). The supernatants containing peptides and small proteins were dried in SpeedVac. Dried supernatants were dissolved in 0.1% formic acid, and protein concentrations were determined with NanoDrop. In order to be able to detect also the possible small proteins and natural peptides in the venom samples, and not only tryptic peptides, each supernatant sample was divided into two portions. One portion of each sample was run with HPLC-MS/MS without carrying out digestion before the analysis. In order to analyse the tryptic peptides, the second portion of each sample was in-solution digested by reconstituting the sample to six M urea in 50 mM Tris-HCl (urea was used to denaturate the protein structure), reduced with 200 mM 1,4-dithiotreitol, alkylated using 200 mM iodoacetamide, and digested with trypsin (1:30 trypsin:substrate ratio) overnight at 37 °C. After digestion proteins were desalted using Sep-Pak C18 cartridges (Waters Corporation, Milford, MA, USA) and dried in SpeedVac.

### 5.4. LC-ESI-MS/MS Analysis

The tryptic peptide samples were then re-suspended into 0.1% formic acid so that a theoretical protein concentration was 3 µg/µL for each sample. The samples were further diluted in a ratio 1:15, so that theoretically 200 ng of each sample was injected into a nanoflow HPLC system (Easy-nLC1200, Thermo Fisher Scientific, Bremen, Germany) coupled to the Q Exactive HF (Thermo Fisher Scientific, in-gel digested samples) or to the Q Exactive mass spectrometer (Thermo Fisher Scientific, in-solution digested samples and concentrated gel samples) equipped with a nano-electrospray ionisation source. Peptides were first loaded on a trapping column and subsequently separated inline on 15 cm C18 column (75 µm × 15 cm, ReproSil-Pur 5 µm 200 Ã C18-AQ, Dr. Maisch HPLC GmbH, Ammerbuch-Entringen, Germany); the mobile phase consisted of water with 0.1% formic acid (solvent A) and acetonitrile/water (80:20 (*v*/*v*)) with 0.1% formic acid (solvent B); the active gradient lengths used depended on the complexity of the sample ranging between 10 to 120 min. The flow rate was set at 300 nL/min. Concentrated gel samples were first run using data-dependent acquisition method. In order to increase the number of identifications, precursor ions of identified peptides were added to mass exclusion list, and samples were re-run with mass spectrometer. This was repeated once. When creating the exclusion lists, precursor mass tolerance was set to ± 8 ppm, fragment mass tolerance to at ± 0.02 Da, maximum missed cleavages was set to two. Only precursors with high or medium confidence were selected for the exclusion lists. A significance threshold of *p* < 0.05 was used. No replicate runs were made on other samples.

### 5.5. Data Analysis

For data analysis, raw data were processed using Proteome Discoverer 2.2 (Thermo Fisher Scientific). The software was connected to an in-house server running the Mascot 2.6.1 software (Matrix Science). Data were searched against a custom-made venom database and target decoy database. The venom database was downloaded from UniProt (28.6.2018) and contained 6328 protein sequences from 655 different venomous species (See Table S4). In Proteome Discoverer MS/MS peptide mass tolerance was set at ± 8 ppm, fragment mass tolerance at ± 0.02 Da and the maximum missed cleavages was set to two. Carbamidomethyl (C) was chosen as a static modification. Oxidation (M) and acetyl (protein N-terminus) were chosen as dynamic modifications. Trypsin was selected as enzyme for digested samples. The non-digested samples were searched with ‘none’ selected as the enzyme. The Fixed Value, PSM validator node, was used to assign confidence to PSMs based on fixed score threshold of 0.05. Only protein with high or medium confidence are listed in this paper, and all identifications are based on at least two peptides per protein. A significance threshold of *p* < 0.05 was used. The processed data were exported as Microsoft Excel files and proteins which were identified with only one peptide were filtered out from the results and those that did not match the database were not identified. The mass spectrometry proteomics data have been deposited to the ProteomeXchange Consortium via the PRIDE [47] partner repository with the dataset identifier PXD020411.

## Figures and Tables

**Table 1:** This table summarises some of the common protein families found in various snake venoms, including elapids.

Protein Family	Short Description	Mode of Action/Function	MW (Kda)	References
PLA_2_	Esterases with a unique pancreatic loop at 62–66 residues.	Cleaves the sn-2 acyl bond to hydrolyse phospholipids to release fatty acid and lyso-phospholipid.	13–15	[24,25]
3FTxs	Non-enzymatic polypeptides consisting of 60–74 amino acids, of which three peptide loops are stabilised by four or five disulphide bridges.	Mainly function as potent inhibitors of neuromuscular transmission, cardiac function and acetylcholinesterase. Moreover, interfere with nicotinic and muscarinic acetylcholine receptors, acetylcholinesterase, ion channels, and cell membranes.	6–9	[26,27,28,29,30]
SVMPs	Monozinc endopeptidases.	Not well understood, but in general, they induce haemorrhage, inflammation, and apoptosis, while inhibiting aggregation of platelets.	20–100	[31,32]
KUN	Dendrotoxins consisting of 57–60 amino acids organised in a single polypeptide chain cross-linked by three disulfidebridges.	Facilitate the release of the neurotransmitter acetylcholine at the neuromuscular junction, resulting in blockage of certain voltage-dependent K+ channels in the nerve presynaptic terminals.		[33]
LAAO	Flavoenzymes found in acidic, neutral and basic forms.	Not well understood but exist as homodimers which contribute the cytotoxic activities.	50–70	[34]
CRiSP	Single chain polypeptides containing 16 cysteine molecules, of which ten are clustered in the C-terminal of the sequence.	Act as inhibitors of cyclic nucleotide-gated channels and L-type Ca2+ and/or K+ channels, to prevent smooth muscle contraction.	20–30	[35,36,37]
NPs	38 residue peptides with four proline residues, of which three are clustered in the C terminal of the molecule.	Diffuse into vascular smooth muscle cells to activate guanylate cyclase inducing vasodilation.	–	[38,39]

Abbreviations: Phospholipase A2 (PLA_2_); Three finger toxins (3FTxs); Snake Venom Metalloproteinases (SVMPs); kunitz peptides (KUN); L-amino acid oxidases (LAAO); Cysteine-rich secretory proteins (CriSP); Natriuretic Peptides (NPs).

**Table 2:** This table shows the number of proteins identified per approaches for the venoms of *Naja mossambica* and *Naja nigricincta. nigricincta.*

Method Approach	Number of Proteins Per Species	
	*N. mossambica* n (%)	*N. n. nigricintca* n (%)
In-gel digestion (Silver-stained)	71 (94.7)	68 (93.2)
In-gel digestion (Not stained)	54 (72.0)	58 (79.5)
In solution digestion	32 (42.7)	22 (30.1)
No digestion	4 (5.3)	3 (4.1)

**Table 3:** This table summarises the number of proteins identified for each protein family from the venoms of *N. mossambica* and *N. n. nigricincta.*

Protein Family	Number of Proteins		
	*N. mossambica*	*N. n. nigricintca*	Common to Both Snakes
5’-nucleotidase	0	1	1
Cathelicidin	2	0	0
Cystatin	1	0	1
Cysteine-rich secretory protein (CRiSP)	1	3	6
Flavin monoamine oxidase	1	1	4
Glycosyl hydrolase 56	0	2	0
Nerve growth factor (NGF-Beta)	0	1	3
Nucleotide pyrophosphate/phosphodiesterase	0	0	1
Ohanin/vespryn	0	0	1
Peptidase S1	0	0	1
Peroxiredoxin	0	1	0
Phospholipase A2 (PLA2)	1	1	6
Phospholipase B	0	0	2
Three finger toxins (3FTx)	10	5	20
True venom lectin	0	0	1
Venom complement C3 homolog	0	0	3
Venom Kunitz-type family	1	0	0
Venom metalloproteinase M12B (SVMP)	1	1	7
Total	18	16	57

The number of proteins identified by four different approaches was pooled together to classify them into their respective families for *N. mossambica* and *N. n. nigricincta* venoms.

## Supplementary Materials

The following are available online at https://www.mdpi.com/2072-6651/12/8/520/s1, Table S1: Proteins identified from the venoms of *N. n. nigricincta* and *N. mossambica*, Table S2: Total number of proteins found in *N. mossambica*, Table S3: Total number of proteins found in *N. n. nigricincta*, Table S4: List of species in the custom-made venom database.
